# Vascular endothelial growth factor expression and inhibition in uveal melanoma cell lines

**DOI:** 10.3332/ecancer.2013.336

**Published:** 2013-07-31

**Authors:** Patrick Logan, Julia Burnier, Miguel N. Burnier

**Affiliations:** The Henry C. Witelson Ocular Pathology Laboratory, McGill University, Montreal, Quebec H3A 2B4, Canada

**Keywords:** uveal melanoma, VEGF-A, invasion, migration, proliferation, bevacizumab

## Abstract

**Background:**

Uveal melanoma (UM) is a disease that affects approximately five people per million in the United States. This disease metastasises predominantly to the liver, and treatment options following the clinical detection of these sequelae are limited. Vascular endothelial growth factor-A (VEGF-A) is the primary activator of tumour angiogenesis and functions by binding to VEGF-Receptor 2 (VEGF-R2) and is often required for tumour growth beyond 2–3 mm. The purpose of this study was to investigate the expression of VEGF-A and the primary VEGF-R2 in three UM cell lines. Furthermore, we investigated the effects of VEGF-A inhibition on receptor activation and production of other cytokines. Finally, the effects of VEGF-A inhibition on the proliferation, migration, and invasion in the cell lines were ascertained.

**Materials:**

Three UM cell lines (92.1, OCM-1, and UW-1) were incubated with and without the addition of 100 μg/mL of bevacizumab. VEGF-A expression under both conditions was determined by sandwich enzyme-linked immunosorbent assay (ELISA), and phosphorylated VEGF-R2 expression was determined using western blot. The effects of VEGF-A inhibition on 20 cytokines (IL-1a, IL-2, IL-5, IL-8, IL-12p70, GM-CSF, IFNy, CCL3, MMP-9, TNF-a, IL-1b, IL-4, IL-6, IL-10, IL-13, GRO, MCP-1, MIP-1b, and RANTES) were determined using a multiplex sandwich ELISA. Proliferation rates before and after treatment were evaluated via sulforhodamine B assay, and migration and invasion assays implementing the Boyden chamber technique, the latter with artificial extracellular matrix, were used to assess their respective abilities. The Student’s t-test was used to compare changes in cytokine expression following VEGF-A inhibition. Analysis of variance was used to compare changes in the functional abilities of three uveal melanoma cell lines following VEGF-A inhibition. A *P*-value < 0.05 was considered statistically significant.

**Results:**

All three cell lines produced copious amounts of VEGF-A in culture (92.1, 11785.5 ± 231.8 pg/μL; OCM-1, 4608.0 ± 324.0 pg/μL; UW-1, 8309.3 ± 634.5 pg/μL), which was reduced to undetectable levels following the administration of bevacizumab (*P*< 0.05). Similarly, detectable phosphorylated VEGF-R2 was present in all cells, which was reduced significantly in all cell lines following bevacizumab treatment (107525.2 ± 8602.0 versus 1024.5 ± 98.2, 46587.3 ± 4192.9 versus 12821.1 ± 1666.7, and 60394.3 ± 4026.4 versus 6908.2 ± 607.2; 92.1, OCM-1, and UW-1, respectively; *P*< 0.05). Of the cytokines investigated, only MMP-9 and CCL3 were ubiquitously altered across all three cell lines following bevacizumab treatment; they were upregulated (CCL3: 1072.50 ± 18.77 pg/mL versus 1281.00 ± 72.34 pg/mL; 22.5 ± 7.85 pg/mL versus 62.00 ± 9.16 pg/mL; 20.33 ± 6.35 pg/mL versus 35.00 ± 6.22 pg/mL; control versus bevacizumab; MMP-9: 25.50 ± 5.47 pg/mL versus 88.25 ± 13.38 pg/mL; 19.75 ± 4.14 pg/mL versus 45.25 ± 8.36 pg/mL; 3.25 ± 1.09 pg/mL versus 19.25 ± 3.77 pg/mL; control versus bevacizumab; 92.1, OCM-1, and UW-1, respectively; *P*< 0.05). Bevacizumab significantly reduced the proliferation of one cell line (92.1: 0.405 ± 0.012 versus 0.509 ± 0.033; bevacizumab versus control; values OD; *P*< 0.05), the migration of two cell lines (92.1: 0.071 ± 0.003 versus 0.115 ± 0.003; OCM-1: 0.049 ± 0.005 versus 0.117 ± 0.014; bevacizumab versus control; values OD; *P*< 0.05), and did not significantly affect invasion.

**Conclusion:**

Despite the significant reduction in phosphorylated VEGF-R2 levels, bevacizumab did not have a dramatic impact on the functional abilities of the three UM cell lines studied. Our results indicate that compensatory mechanisms, such as the upregulation of MMP-9 and CCL-3, following bevacizumab administration may mitigate its effects on these abilities.

## Background

Uveal melanoma (UM) is a malignancy of the uveal tract, which is composed of the pigmented portions of the eye: the iris, the ciliary body, and the choroid. Of these anatomical structures, the choroid is the most common location from which UMs arise, accounting for roughly 85% of cases [1]. UM is a rare entity affecting approximately 4.3 people per million in the United States, the vast majority of which are Caucasian males [2]. Despite its paucity, UM is the most common primary intraocular tumour in adults.

Approximately 40% of people with UM will develop metastases within 5–10 years of diagnosis [3, 4]. In the unfortunate instances in which metastases arise, the majority occur in the liver, and 90% of patients will succumb to these sequelae in less than three months [5–8].

Despite technical advances in treating the primary tumour, the incidence of metastasis has remained relatively unchanged over the past four decades [9]. Unlike the primary tumour, for which many efficacious and conservative treatments are available, limited and markedly less successful treatments for overt liver metastases exist. In fact, there are no guidelines or standard treatments for liver metastasis derived from UM tumours; the typical systemic chemotherapy agents have been tried and met with limited success [10]. Although surgical resection can increase the life span of those suffering from liver metastasis, the tendency for multiplicity and diffusivity of UM liver metastases renders this treatment applicable for only a small percentage of patients [8, 11–13].

Vascular endothelial growth factor (VEGF) is the most potent angiogenic stimulator in mammals. First identified in 1971 by Folkman *et al*, this signalling molecule is involved in both physiological and pathological angiogenesis [14–17]. VEGF fundamentally describes a family of five homodimer signalling molecules that include VEGF-A through VEGF-D and placental growth factor (P1GF) [18]. Although all of these molecules influence elements of the angiogenic process to differing degrees, including blood vessel lumen formation and endothelial cell migration and proliferation, VEGF-A is commonly branded with the generic term VEGF, as it is responsible for the majority of the physiological functioning of this family [18].

Physiologically, when a tumour grows larger than 2–3 mm in diameter, it requires oxygen and nutrients for further efficacious proliferation [19]. This juncture, dubbed the angiogenic switch, has therefore been a focus of researchers of nearly every variety of solid tumour. The allure of VEGFs and their receptors as potential therapeutic targets for cancer treatment is compounded by the knowledge that these receptors are typically inactive during normal physiological conditions, and thus their inhibition is generally well tolerated [20]. Furthermore, the consequence of VEGF signalling in a variety of tumours extends beyond merely recruiting endothelial cells and forming perfusion channels; signalling through both VEGF-Receptor 1 (VEGF-R1) and VEGF-Receptor 2 (VEGF-R2) has been implicated in cancer progression, together contributing to tumour cell invasion, migration, proliferation, and metastasis.

Recent studies have shown that patients with UM have elevated levels of VEGF-A in both the aqueous humour and the vitreous humour [21]. In the aqueous humour, increased VEGF-A production is attributed to both the retina and tumour cells and thus presents as a potential therapeutic target [22]. *In vitro*studies have also revealed that UM cell lines produce VEGF-A in normal culture and production increases under hypoxic conditions [23, 24]. Furthermore, in an animal model of UM, increased VEGF-A levels were detectable in serum, and these escalations correlate with increased metastasis [25]. Reports vary with regard to VEGF-A positivity in primary UM tumours, ranging from 22% to 84%, and there are conflicting reports regarding correlations between expression levels and tumour size, vascularisation, or metastasis [26, 27].

Based on the importance and implications of VEGF-A in both local and metastatic growth in a wide spectrum of malignancies, it is unsurprising that several pharmaceutical VEGF inhibitors have been developed. The first anti-angiogenic drug, a monoclonal antibody targeting all isoforms of VEGF-A named bevacizumab, was approved in 2003 for the treatment of metastatic colorectal cancer [28].

Unsurprisingly, anti-VEGF therapies have also been explored for the treatment of UM. In 2010, Yang *et al*demonstrated that bevacizumab reduced intraocular tumour size and the number of micrometastases in a short-term mouse model of UM [29]. A clinical trial using intravitreal bevacizumab injections to reduce the size of UM tumours is currently underway [30]. Attributable to the aforementioned appeal of using bevacizumab’s vessel stabilising properties in conjunction with other pharmaceutical agents, several clinical trials using combination therapies are also being conducted [31].

Based on the effectiveness of anti-angiogenic therapy for several cancers, we assessed whether three UM cell lines produced VEGF-A in culture and whether they also produced the primary receptor for this ligand, VEGF-R2. We also sought to determine whether the VEGF-R2 was activated by auto-or paracrine signalling and whether this process could be abrogated using the commercially available VEGF-A inhibitor, bevacizumab. Moreover, we investigated the effects of VEGF-A inhibition on a large panel of complementary cytokines to determine whether they are upregulated to offset the reduced receptor tyrosine kinase activity. Finally, we determined how inhibiting VEGF-A affects the ability of our cell lines to proliferate, migrate, and invade.

## Materials

### Cell lines and culture

Three UM cell lines, 92.1 (Dr. Jager; University Hospital Leiden, The Netherlands), OCM-1 (Dr. Albert; University of Wisconsin-Madison, United States), and UW-1 (Dr. Albert), were used in this study. The functional characteristics of these cell lines are described elsewhere [32–34]. During normal culture periods, cells were incubated at 37°C in a humidified 5% CO_2_-enriched atmosphere (Thermo Forma Series II Water Jacketed CO_2_Incubator; Fisher Scientific Limited, Ontario, Canada). Cells were cultured in RPMI 1640 medium (Invitrogen, Ontario) that was supplemented with 5% heat-inactivated foetal bovine serum (FBS; Invitrogen), 1% fungizone (Invitrogen), and 1% penicillin-streptomycin (Invitrogen) as a monolayer in 25-cm^2^flasks (Fisher) and observed twice weekly, at every media change, for normal growth by phase contrast microscopy. Cell viability was determined using the trypan blue exclusion test [35]. For experiments requiring serum-starved cells, the existing culture media were removed, and the cells were washed in phosphate-buffered saline (PBS) and recultured using identical media constituents and conditions excluding the 5% FBS for 24 h prior to experimentation.

### Multiplex sandwich ELISA

To determine whether the three cell lines produced VEGF and other pro-angiogenic cytokines, we used commercially available multiplex sandwich enzyme-linked immunosorbent assays (ELISAs). We also investigated whether the administration of bevacizumab affected the production of these factors using ELISA.

First, 500,000 cells from each cell line were cultured separately in six-well plates with 5% FBS supplemented media. Cells were allowed 24 h to adhere before the serum media were removed. Next, the cells were washed twice with PBS, and serum-free media were added to the wells. For the experimental condition, 100 μg/mL of bevacizumab (Avastin^®^; Roche; Laval, Quebec, Canada) was also added to the wells. Twenty-four hoursafterchangingtoserum-freemediawithorwithouttheadditionofbevacizumab,500μLofconcentrated,conditionedmediawasextracted from each well for multiplex sandwich ELISA analysis (Quantibody^®^Human Angiogenesis Array I and Human Cytokine Array 1; RayBiotech, Inc., Norcross, Georgia, United States). For this experiment, we used two different platforms, which provide quantitative data pertaining to ten pro-angiogenic cytokines (angiogenin, ANG-2, EGF, bFGF, HB-EGF, HGF, Leptin, PDGF-BB, P1GF, and VEGF) and 20 cytokines (IL-1a, IL-2, IL-5, IL-8, IL-12p70, GM-CSF, IFNy, CCL3, MMP-9, TNF-a, IL-1b, IL-4, IL-6, IL-10, IL-13, GRO, MCP-1, MIP-1b, RANTES, and VEGF). The ELISA experiments were performed per the manufacturer’s instructions. After completing the experimental procedures, slides were sent to RayBiotech, Inc., for fluorescent analysis with the Quantibody^®^Q-Analyzer software. All conditions were repeated in quadruplicate and normal; unconditioned media both with and without the addition of 100 μg/mL of bevacizumab were used as negative controls.

### Western blot

Next, to determine whether VEGF-R2 was activated by endogenous VEGF-A, a western blot for the phosphorylated version of the protein was performed. This process was executed on control cells and those in which VEGF-A was inhibited with 100 μg/mL bevacizumab. The cells were grown in a six-well plate with 5% supplemented serum media to a total density of 500,000 cells, after which they were grown in serum-free media for 24 h prior to protein extraction. The experimental group was incubated with 100 μg/mL of bevacizumab (Roche) for 24 h prior to protein extraction. Next, protein was extracted from the pellets using complete Protease Inhibitor Cocktail Tablets (Roche), PhosSTOP Phosphatase Inhibitor Tablets (Roche), and lysis buffer (150 mM NaCl, 50 mM HEPES, pH 7.4, 1 mM EGTA, 1 mM Na_3_VO_4_, 10 mM NaF, 1 mM phenylmethylsulfonyl fluoride, 5% glycerol, and 1% Triton X-100) per the manufacturer’s instructions. After extraction, protein quantification was determined using the Micro BCA Protein Assay Kit (Thermo Scientific, Rockford, Illinois, United States) per the manufacturer’s instructions and read using the Bio-Tek EL-800 Universal plate reader (Bio-Tek Instruments, Winooski, Vermont, Unites States) at a wavelength of 560 nm. Protein from all samples was standardised to 15 ng/μL. Next, 30 μL of control and experimental samples were subjected to sodium dodecyl sulfate polyacrylamide gel electrophoresis on 8% acrylamide separating gel before being transferred to nitrocellulose membranes (Invitrogen) and incubating overnight at 4°C with Phospho-VEGF-R2 (1:500; Cell Signalling Technology, Beverly, Massachusetts, United States) and β-Actin (1:1000; Abcam plc, Cambridge, Massachusetts). The film was developed using the Pierce ECL Western Blotting Substrate (Thermo Scientific) and Kodak Biomax XAR film (Kodak, Rochester, New York, United States), per manufacturer’s instructions; the film was exposed for 30 s. For negative controls, the aforementioned process was repeated to save omission of the primary antibody.

To objectively compare activated VEGF-R2 protein levels between control cells and those treated with bevacizumab, the optical density (OD) of the positive bands from the western blot was analysed using ImageJ Software [version 1.46d; US National Institutes of Health (NIH)] [36, 37]. After scanning the film and generating a JPG image, lanes were individually selected using the Analyze > Gels command. Next, plots of the relative densities of the content of each lane were determined, and the data from each plot area were calculated and exported to Microsoft Excel (2010). This process was repeated for every control and experimental condition for both β-actin and Phospho-VEGF-R2. To normalise all lanes for loading discrepancies, the densest (highest value determined by ImageJ) β-actin lane was given a relative value of 1, and all other β-actin values were normalised to the density of this lane. Next, all phospho-VEGF-R2 bands were normalised according to their respective loading controls. Finally, relative expression of VEGF-R2 before and after bevacizumab treatment was determined for each cell line by dividing the normalised OD of the control by the normalised OD of the experimental condition.

### Quantitative real-time polymerase chain reaction

To ascertain the effects of bevacizumab on the transcription rate of VEGF-A, quantitative real-time polymerase chain reaction (RT-qPCR) was performed. The three cell lines were grown in six-well plates in regular culture media until they reached a total of 500,000 cells per well. Twenty-four hours before harvesting, supplemented media were replaced with serum-free media, and the experimental group was augmented with 100 μg/mL of bevacizumab. The cells were harvested, and RNA was extracted using the Qiagen RNeasy kit (Qiagen, Mississauga, Ontario) per the manufacturer’s instructions. Briefly, cells were disrupted and homogenised using lysate buffer, and after mechanical separation using a syringe, insoluble material was removed by centrifugation. The lysate solution was mixed with one volume of 70% ethanol before adding the solution to RNeasy minicolumns, which were centrifuged and then washed twice using the included buffer solutions. Finally, total cellular RNA was eluted using RNase-free water.

The RNA was then converted to a cDNA library using iScript™ Reverse Transcription Supermix for RT-qPCR (Bio-Rad Laboratories, Hercules, California, United States) per the manufacturer’s instructions. After adding the Supermix to each RNA sample, a Chromo4 thermocycler (MJ Research, Waltham, Massachusetts) was used to incubate the mixture with the following settings: priming, 5 m at 25°C; reverse transcription, 30 m at 42°C; and RT inactivation, 5 m at 85°C. All future experiments used the cDNA generated using the techniques above. For negative controls, the process was repeated without the addition of the reverse transcription.

The iQ™ SYBR^®^Green Supermix (Bio-Rad Laboratories) was then added to the cDNA from each sample. Each sample was prepared in triplicate using β-actin, VEGF-A, CCR1 (the primary receptor for CCL3), MMP-9, and CCL3 primers (QuantiTect^®^Primer Assay; Qiagen) and the following settings were programmed into the Opticon Monitor software (Bio-Rad Laboratories) and run using the Chromo4 thermocycler (MJ Research): 15 m at 95°C, 15 s at 94°C, 30 s at 55°C, 30 s at 72°C, followed by a plate read. This process was repeated a total of 40 times.

For all comparisons between the treated and untreated cells, the Pfaffl fold-change method was used [38]. This method was selected as it accounts for potential differences in primer efficiency and non-perfect transcript doubling. First, standard curves were generated using the two primers (β-actin [Invitrogen] and VEGF-A [Invitrogen]) and known quantities of RNA, and from this standard curve, the slope of the line was calculated.

### Functional assays

In order to determine the effects of various treatments on the functional abilities of UM cells, we compared the controls with those treated with 100 μg/mL of bevacizumab. The goal of the experiments was to determine what effect bevacizumab has on the functional abilities.

### Proliferation assay

Cell proliferation was calculated using the sulforhodamine-B-based *in vitro*toxicology assay kit (TOX-6; Sigma, St. Louis, Missouri, United States), executed per the manufacturer’s instructions. Sulforhodamine B stains cellular proteins, and thus the amount of dye incorporation is an indirect measure of total biomass and, consequently, cell number [39]. Briefly, 25,000 cells from each condition were grown in 96-well plates in serum-free media. For experimental conditions, 100 μg/mL of bevacizumab was added to the wells. After 24 h, cells were fixed using trichloroacetic acid for one hour before washing with water. Next, sulforhodamine B solution was added to the cells, and they were allowed to stain for 30 min. After residual solution was removed by washing, dye was solubilised with the provided Sulforhodamine B Assay Solubilisation Solution, and wells were agitated for 5 min. After visual confirmation that the dye was dissolved, the absorbance of the wells was determined at 565 nm using the Bio-Tek EL-800 Universal plate reader. Data were collected using KC Junior software (Bio-Tek). All experiments were repeated in triplicate. Media with and without added bevacizumab were used as controls.

### Migration assay

Conceptually, the migration assay functions by inserting serum-starved cells into an upper chamber that has a porous, membrane base. With the addition of a chemoattractant in the lower chamber, cells will migrate towards the attractant, and after staining, the number of migrated cells can be counted and compared between conditions.

Migration assays were performed using the QCM™ 24-well Colorimetric Cell Migration Assay (EMD Millipore, Billerica, Massachusetts) per the manufacturer’s instructions. Briefly, 300,000 cells were serum starved for 24 h before adding them in serum-free media into the upper chamber. For a chemoattractant, serum supplemented media were added to the lower. For experimental conditions, 100 μg/mL of bevacizumab was added to the upper chamber with the cells. The cells were then placed back into the incubator under normal culture conditions to allow migration. After 24 h, the non-migrating cells from the upper chamber were removed by pipetting, and the lower portion of the upper chamber was incubated in cell stain for 20 min. Residual, non-migrating cells from the upper chamber were removed by swabbing. The bottom of the chamber, containing the stained, migrated cells, was incubated in extraction buffer. After transferring 100 μL of this solution to a 96-well plate, the OD at 560 nm was determined using the Bio-Tek EL-800 Universal plate reader. All experiments were repeated in triplicate. Media with and without added bevacizumab were used as controls.

### Invasion assay

The Colorimetric QCM ECMatrix Cell Invasion Assay (EMD Millipore) was used to determine the invasive ability of UM cell lines. This assay functions similarly to the migration assay described above; the cells were prepared in the same manner and the technical aspects of the kit are identical. However, while the migration assay has a membrane with 8-μm holes for the cells to migrate through, in the invasion assay, these pores are covered with a basement membrane (ECMatrix). This membrane is designed to represent the extracellular matrix present *in vitro;*in this context, the cells must actively degrade this membrane in order to migrate into the lower chamber, in a manner mimicking growth in an organ. Only cells that accomplish this task will be subsequently detected by the plate reader. Media with and without added bevacizumab were used as controls.

### Statistical analysis

To compare between multiple means, such as for the multiple conditions used in the proliferation, migration, and invasion assays and for comparing CCL3 and MMP-9 protein secretion, we first determined homogeneity of variance using Levene’s statistic. If Levene’s statistic was > 0.05, we performed a one-way analysis of variance (ANOVA). To compare experimental conditions to the controls, Dunnett’s two-sided tests were used for post-hoc analysis. Analyses were performed using SPSS version 20 (IBM Corporation). The Student’s t-test was used to determine differences for all tests requiring the comparison of only two means (Microsoft Excel).

For all statistical analyses, a *P*-value of < 0.05 was considered statistically significant. All data are presented as mean ± standard deviation (SD).

## Results

### Cytokine expression in UM cell lines before and after VEGF-A inhibition

**Figure 1:**
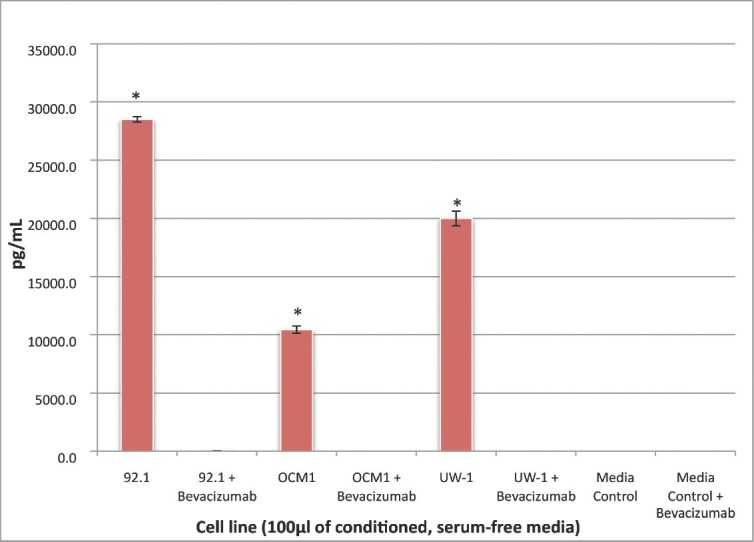
Figure 1: VEGF-A secretion by three UM cell lines before and after bevacizumab treatment: secretion of VEGF-A by the three UM cell lines (92.1, OCM-1, and UW-1) in conditioned media before and after the administration of 100 μg/mL of bevacizumab, as determined using a sandwich ELISA. Normal media with and without bevacizumab were used as controls. *P < 0.05; Student’s t-test.

All three cell lines produced VEGF-A in culture (mean ± SD): 92.1, 11785.5 ± 231.8 pg/μL; OCM-1, 4608.0 ± 324.0 pg/μL; UW-1, 8309.3 ± 634.5 pg/μL. After bevacizumab administration, there was no detectable VEGF-A in any cell line, a difference that was significant (*P*< 0.001) for all (Figure 1).

With respect to the pro-angiogenic cytokines, angiogenin was significantly reduced in the OCM-1 and UW-1 cell lines following bevacizumab treatment (1562.0 ± 16.9 pg/μL versus 1401.2 ± 16.2 pg/μL and 4980.9 ± 187.6 pg/μL versus 1736.8 ± 101.1 pg/μL, respectively; Student’s t-test; *P*< 0.05, for both). EGF was significantly reduced following bevacizumab treatment of the UW-1 cell line (33.3 ± 2.0 pg/μL versus 23.7 ± 2.4 pg/μL, respectively; Student’s t-test; *P*< 0.05). Similarly, both HB-EGF and P1GF were significantly reduced in the UW-1 cell line after bevacizumab administration (366.4 ± 47.3 pg/μL versus 59.1 ± 4.2 pg/μL and 64.6 ± 4.4 pg/μL versus 22.1 ± 2.1 pg/ μL, respectively; Student’s t-test; *P*< 0.05, for both). No significant expression changes in any other cytokines were noted after bevacizumab treatment (data not shown). Although not significant, it is of note that bevacizumab induced an increase in EGF and HB-EGF in the OCM-1 cell line.

For the cytokine array, of the 20 cytokines tested, only four were significantly affected following bevacizumab treatment: IL-4, GM-CSF, CCL3, and MMP-9, the former two upregulated and the latter two downregulated. IL-4 secretion was significantly reduced in the 92.1 and OCM-1 cell lines after bevacizumab treatment (223.00 ± 35.44 pg/mL versus 124.33 ± 16.74 pg/mL; 213.50 ± 31.93 pg/mL versus 114.25 ± 19.03 pg/mL, respectively; Student’s t-test; *P*< 0.05, for both). GM-CSF secretion was also significantly reduced in the OCM-1 cell line following bevacizumab therapy (167.00 ± 11.43 pg/mL versus 109.50 ± 21.01 pg/mL; control versus bevacizumab; Student’s t-test; *P*< 0.05).

**Figure 2:**
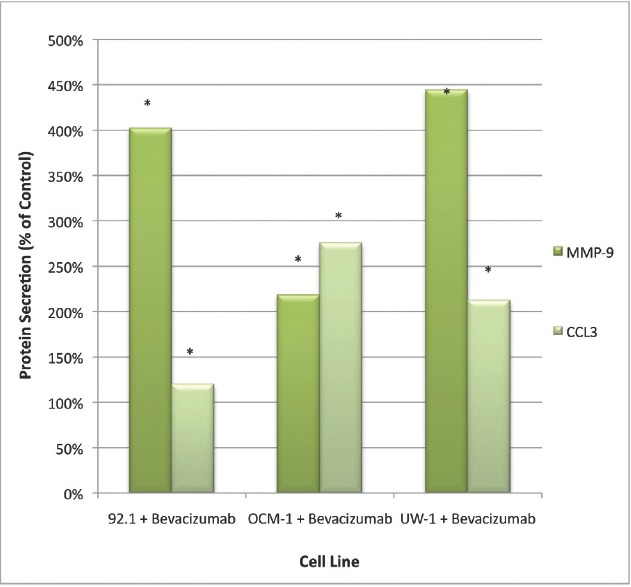
Figure 2: MMP-9 and CCL3 protein secretion by UM cell lines: secretion of CCL3 and MMP-9 by three UM cell lines following treatment with 100 μg/mL of bevacizumab. Results are shown as percentage of control. *P < 0.05; Student’s t-test (compared with control). Bars: standard deviation.

Only CCL3 and MMP-9 were significantly upregulated following bevacizumab treatment across all three cell lines. For CCL3, the values were 1072.50 ± 18.77 pg/mL versus 1281.00 ± 72.34 pg/mL; 22.5 ± 7.85 pg/mL versus 62.00 ± 9.16 pg/mL; 20.33 ± 6.35 pg/mL versus 35.00 ± 6.22 pg/mL; control versus bevacizumab; 92.1, OCM-1, and UW-1, respectively; Student’s t-test; *P*< 0.05, for all (Figure 2).

For MMP-9, the values were 25.50 ± 5.47 pg/mL versus 88.25 ± 13.38 pg/mL; 19.75 ± 4.14 pg/mL versus 45.25 ± 8.36 pg/mL; 3.25 ± 1.09 pg/mL versus 19.25 ± 3.77 pg/mL; control versus bevacizumab; 92.1, OCM-1, and UW-1, respectively; Student’s t-test; *P*< 0.05, for all (Figure 2).

There was no detectable VEGF-A in serum-free media with or without the addition of bevacizumab.

### VEGF-R2 activation before and after VEGF-A inhibition

**Figure 3:**
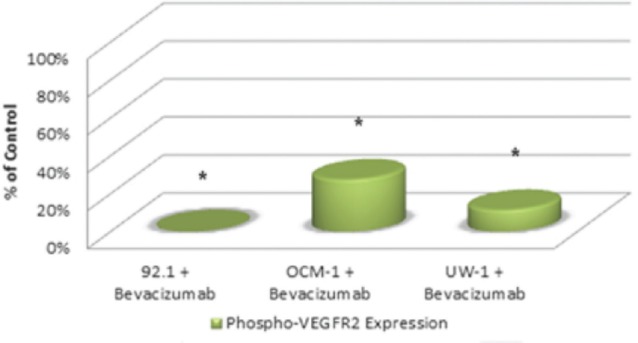
Figure 3: Phosphorylated VEGF-R2 expression in three UM cell lines following bevacizumab treatment as determined by western blot. Expression of phosphorylated-VEGF-R2 before and after bevacizumab treatment is expressed as a percentage of the control. The OD of the western blots was quantified using ImageJ software (NIH). *P< 0.05; Student’s t-test.

Activated VEGF-R2 was detectable in all three cell lines under control conditions. The ODs after correcting for loading bias were as follows: 92.1 = 107525.2 ± 8602.0, OCM-1 = 46587.3 ± 4192.9, and UW-1 = 60394.3 ± 4026.4. After bevacizumab administration, activated VEGF-R2 levels significantly reduced to 1% (1024.5 ± 98.2), 28% (12821.1 ± 1666.7), and 14% (6908.2 ± 607.2) of the 92.1, OCM-1, and UW-1 controls, respectively (Student’s t-test; *P*< 0.05; Figure 3).

### Changes in cytokine mRNA following VEGF-A inhibition

Inhibition of VEGF-A with 100 μg/mL of bevacizumab caused a slight yet consistent upregulation of VEGF-A across all three cell lines. VEGF-A mRNA increased 1.14, 1.18, and 1.37 fold, for the 92.1, OCM-1, and UW-1 cell lines, respectively.

Surprisingly, after bevacizumab treatment, transcription of CCL3 and MMP-9 was not drastically upregulated in any cell line despite the significant changes in protein secretion: fold changes for the 92.1, OCM-1, and UW-1 cell lines were 1.95, 1.08, and 0.97 for CCL3 and 1.48, 1.67, and 1.02 for MMP-9, respectively. Transcripts of the primary CCL3 receptor, CCR1, were detected in all three cell lines and this production increased slightly following bevacizumab treatment (1.20, 1.83, and 1.24-fold increase for 92.1, OCM-1, and UW-1, respectively).

### Changes in proliferation following VEGF-A inhibition

**Figure 4:**
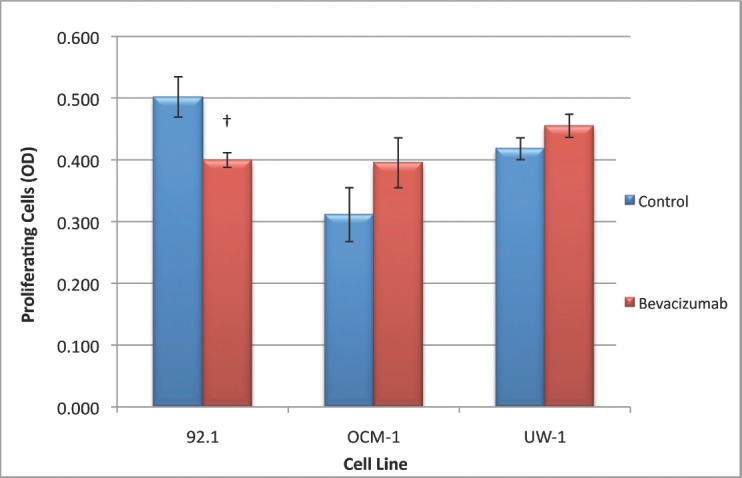
Figure 4: Proliferation of UM cell lines. Graphical representation of the effects of different treatment modalities on the proliferative ability of three UM cell lines. †P < 0.05, Dunnett’s test (compared with control); bars: standard deviation.

For the 92.1 cell line, the ANOVA was statistically significant and it passed the homogeneity of variance test (ANOVA; *P*< 0.05 and Levene’s statistic; *P*> 0.05). Bevacizumab treatment significantly reduced proliferation compared with the control (0.405 ± 0.012 versus 0.509 ± 0.033; bevacizumab versus control; Dunnett’s test; *P*< 0.05; all values OD; Figure 4).

For the OCM-1 cell line, the ANOVA was not statistically significant, but it did pass the homogeneity of variance test (ANOVA; *P*> 0.05 and Levene’s statistic; *P*> 0.05); bevacizumab treatment did not significantly reduce proliferation compared with the control (Figure 4).The ANOVA for UW-1 was also non-significant and complied with homogeneity of variance (ANOVA; *P*> 0.05 and Levene’s statistic; *P*> 0.05); bevacizumab did not significantly reduce proliferation compared with the control (Figure 4).

### Changes in migration following VEGF-A inhibition

**Figure 5:**
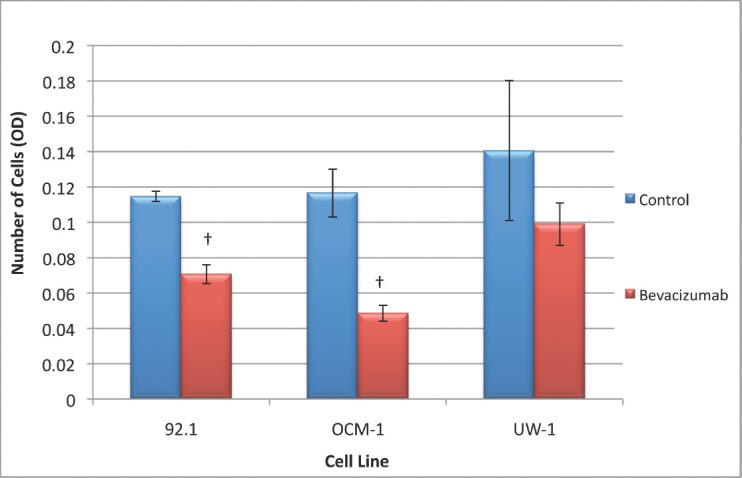
Figure 5: Migration of UM cell lines. Graphical representation of the effects of different treatment modalities on the migratory ability of three UM cell lines. †P < 0.05, Dunnett’s test (compared with the control); bars: standard deviation.

The 92.1 cell migration ANOVA was statistically significant and conformed with the homogeneity of variance (ANOVA; *P*< 0.05 and Levene’s statistic; *P*> 0.05, respectively). Bevacizumab significantly reduced the ability of 92.1 cells to migrate compared with the control (0.071 ± 0.003 versus 0.115 ± 0.003; bevacizumab versus control; Dunnett’s test; *P*< 0.05; values OD; Figure 5).

The ANOVA for OCM-1 was significant and passed the homogeneity of variance test (ANOVA; *P*< 0.05 and Levene’s statistic; *P*> 0.05, respectively); bevacizumab an significantly reduced migration relative to the control (0.049 ± 0.005 versus 0.117 ± 0.014; bevacizumab versus control; Dunnett’s test; *P*< 0.05; values OD; Figure 5).

The ANOVA for the UW-1 cell line was non-significant, and it passed the homogeneity of variance test (ANOVA; *P*> 0.05 and Levene’s statistic; *P*> 0.05); bevacizumab did not significantly reduce migration relative to the control (Figure 5).

### Changes in invasion following VEGF-A inhibition

**Figure 6:**
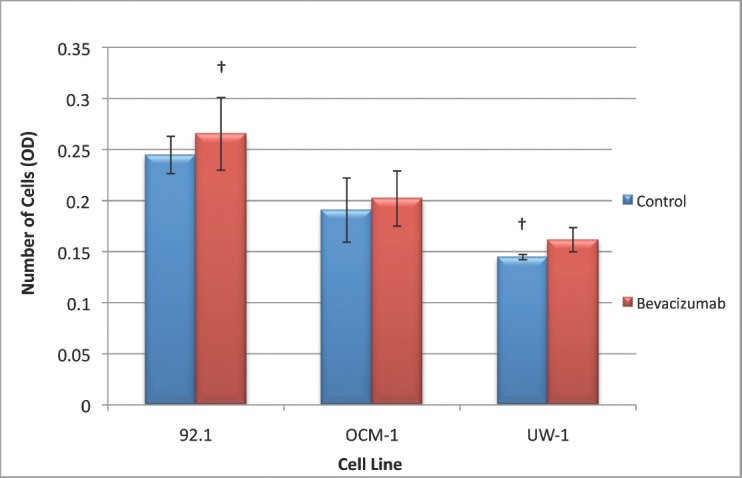
Figure 6: Invasion of UM cell lines. Graphical representation of the effects of different treatment modalities on the invasive ability of three UM cell lines. †P < 0.05, Dunnett’s test (compared with the control); bars: standard deviation.

The ANOVAs for all cell lines were non-significant and passed the homogeneity of variance test (ANOVA; *P*> 0.05 and Levene’s statistic; *P*> 0.05). Therefore, none of the changes in invasion were significant for any cell line; however, all cell lines slightly increased invasion in response to treatment (Figure 6).

## Discussion

Based on recent studies describing the importance of VEGF-A for tumour growth and development, we decided to investigate VEGF-A status in three UM cell lines of varying metastatic potential. Currently, there are limited studies in the literature investigating VEGF-A expression in UM, and fewer assessing the inhibition of this cytokine in animal models, thus comprehensive studies regarding VEGF-A in multiple UM cell lines are needed [25, 26, 29, 40]. In the present study, we determined that three UM cell lines produced copious amounts of VEGF-A (between 4600 and 12,000 pg/μL). It is of note that this exceeds the amount of VEGF-A detected in the serum of patients with UM, which ranged between approximately 40 and 2000 pg/mL [41]. Although direct comparisons between culture experiments and serum from patients should be considered with reservation, our data indicate that the VEGF-A produced by our cell lines might represent a clinically relevant concentration.

All three of the cell lines tested also produced the primary VEGF-A receptor involved in tumour angiogenesis, VEGF-R2, suggesting that both the cytokine and its receptor may be involved in UM development or progression. To better ascertain the functional consequences of this activation, we chose to inhibit VEGF-A using a commercially available inhibitor, bevacizumab. The decision to select bevacizumab over other inhibitors was, in part, based on the fact that bevacizumab is currently the most commonly used anti-angiogenic pharmaceutical used to treat various ocular conditions [42–45]. As expected based on previous reports, bevacizumab did not affect cell cycle or induce cell death at 100 μg/mL [29]. This clinically relevant dosage was capable, however, of completely inhibiting VEGF-A from activating VEGF-R2 in these cell lines. Taken together, these data suggest that UM cell lines use the VEGF-A/VEGF-R2 signalling cascade for at minimum autocrine and paracrine processes, a proceeding that can be abolished with bevacizumab administration.

Autocrine signalling is not uncommon for malignant cells, or even for UM; for instance, it has been shown that the FGF2/FGFR1 autocrine loop induces cell proliferation and survival in UM cells [46]. Furthermore, in many cancers, including ovarian carcinoma, breast cancer, and prostate cancer, VEGF-A signalling can operate in an autocrine manner and induce cell growth [47–49].

We initially considered that UM cells may increase the production of other angiogenic or growth factors in response to bevacizumab treatment, which is the case with colorectal cancer cells after chronic treatment with bevacizumab [50]. This compensatory process was also detected in a study investigating bevacizumab treatment for other ocular conditions in the clinic: bevacizumab therapy induced significant upregulation of IL-8 and TGF-b [51]. However, our data investigating ten pro-angiogenic cytokines do not support this hypothesis, as none of the factors tested were significantly upregulated following bevacizumab administration.

The discrepancy between our *in vivo*data and the *in vitro*data from other studies described above may also be a consequence of a lack of oxygen restrictions in the latter relative to the former; *in vivo*, tumour cells must compete with a host of other cell types for oxygen, whereas in culture, UM cells only compete with other UM cells in a self-limiting manner. In addition, cells grow as a monolayer in culture and thus do not suffer the same surface area-to-volume diffusion restrictions experienced by three-dimensional tumours. Despite the aforementioned discrepancies in oxygen acquisition restrictions between *in vitro*and *in vivo*settings, our experiments were performed in serum-free media, and thus competition for sustenance may be similar between the two cohorts. Therefore, in culture, VEGF-A may be employed more for its growth factor characteristics than for its pro-angiogenic influence, and thus refractory cytokine production following inhibition may reflect growth requirements as opposed to being hypoxia motivated. Therefore, future studies should investigate the compensatory upregulation of growth factors and other cytokines following bevacizumab treatment.

Treating our UM cell lines with bevacizumab successfully inhibited the activation of the primary VEGF-A receptor, VEGF-R2. It was expected that blocking this signal cascade would cause a substantial, global reduction of all functional abilities, as shown in other malignancies, including breast cancer and VEGF-R2+ melanoma cell lines [52, 53]. Our hypothesis was also supported by the positive growth and migratory effects that VEGF signalling imparts on endothelial cells [54]. Surprisingly, despite the abundant and ubiquitous production of both VEGF-A and its receptor, usurping VEGF-A with bevacizumab had only moderate inhibitory effects on the functional capacity of UM cells. Furthermore, VEGF-A inhibition caused a slight, non-significant increase in the invasive ability of our cell lines and yet negatively influenced migration. This is in contrast to a previous study showing that bevacizumab significantly reduced the invasive ability of one human and one mouse UM cell line [29]. While this discrepancy is peculiar, it is of note that the cell line used in the aforementioned study (Mel290) was different from the three used in our study. Due to the heterogeneous nature of malignant cells in general, and in particular UM cells, this result is not entirely surprising and may be related to differing internal signalling pathways. For instance, it has been shown that OCM-1 (a cell line used in the present study) produces high levels of notch signalling intermediates, whereas Mel290 expresses low levels [55].Moreover, it has been demonstrated in bladder carcinoma and glioblastoma that high DII4/Notch signalling may confer resistance to anti-VEGF therapies, thus offering a potential explanation for the aforementioned discrepancy [56, 57].

At first glance, it appears paradoxical that migration was significantly reduced in two cell lines after bevacizumab treatment, and yet invasion was not significantly affected, considering that invasion is a process dependent on the former. A potential explanation is provided by recent evidence demonstrating that autocrine VEGF-A signalling through VEGF-R2 in prostate cells can enhance migration [58]. Thus, in our study, we hypothesise that bevacizumab blocked this signalling cascade that subsequently reduced migration; however, bevacizumab also increased MMP-9 secretion, permitting easier digestion of the basement membrane, which is an inherent impediment to migration in invasion assays [58]. In short, even though our UM cells may be less capable of migrating than controls, they digest the basement membrane more readily, and thus the negative implications of bevacizumab on migration are concealed in the invasion assay.

Based on our data, we were forced to reject the hypothesis that bevacizumab would universally inhibit the functional abilities of our cell lines. Our results strongly suggest that acceptance of the null hypothesis is a consequence of compensatory mechanisms mitigating bevacizumab’s effects, as two cytokines, CCL3 and MMP-9, were significantly upregulated in the cultured media of all cell lines following treatment. MMP-9 is a collagenase responsible for digesting the extracellular matrix, which is a critical process for migration, invasion, and tumour growth *in vivo*. Conceptually, the upregulation and increased secretion of MMP-9 are a plausible reason for the increase in invasive ability that was noted in our study. Although there was no dramatic increase in MMP-9 transcription, it is probable that the increased secretion was a result of post-transcriptional modification, as shown in breast cancer [59]. In the 24-h that these cells were exposed to bevacizumab, we hypothesise that post-translation and secretory changes were activated as initial cellular responses as these processes occur more rapidly than transcriptional alterations. In addition, it has been demonstrated that high MMP-9 expression in UM tumours is associated with poor prognosis [60].

CCL3, also known as macrophage inflammatory protein 1 alpha, binds to two receptors (CCR1 and CCR5) and is responsible for orchestrating acute and chronic inflammatory responses [61]. Studies report that CCL3 is present in some cutaneous melanoma tumours and in the vitreous of eyes with UM; however, to the best of our knowledge, ours is the first evidence that UM cells produce CCL3 in culture [62, 63]. Based on the fact that our cells produce both CCL3 and CCR1 and that CCL3 signalling is an upstream regulator of MMP-9, we hypothesise that the increased secretion in MMP-9 is a consequence of CCL3/CCR1 signalling [64]. Thus, we believe that when the UM cell lines sense a decrease in VEGF-R2 activation following bevacizumab treatment, they respond by upregulating CCL3 secretion. Next, secreted CCL3 activates CCR1 on the host and neighbouring cells, which subsequently increases MMP-9 production. By employing this cascade, cells degrade the extracellular matrix more readily, thereby potentiating dissemination with the ultimate goal of a return to normoxic conditions. It has also been demonstrated that the ECM can harbour biologically active VEGF isoforms; thus, increased MMP-9 production may also release ECM-bound VEGF *in vivo*[65].

*In vivo,*the increased production of CCL3 following bevacizumab treatment may have greater negative effects beyond MMP-9 and invasion augmentation. For instance, CCL3 is a potent macrophage attractant and, in UM, lymphocytic and macrophage infiltration are associated with poor prognosis [66, 67]. In addition, it has been demonstrated that CCL3 enhances VEGF production in macrophages, which further potentiates the compensatory process by which UM cells may counteract a VEGF-A-inhibited environment *in vivo*[68]. Thus, considered anthropomorphically, UM cells produce VEGF for autocrine signalling as well as to facilitate the formation of blood vessels in order to support continual growth. When this process is challenged or inhibited, they respond by upregulating CCL3 production, which re-establishes autocrine signalling and increases MMP-9 production. Amplification of MMP-9, in turn, allows UM cells to degrade the extracellular matrix and escape the VEGF-A inhibited environment as well as releasing any ECM-bound VEGF. In addition, CCL3 attracts macrophages to the tumour where they produce VEGF-A and other cytokines, thus circumventing the VEGF-A usurping.

This study is not the first illustrating that bevacizumab can cause compensatory upregulation of pro-angiogenic and metastatic factors; for instance, colorectal cell lines under chronic bevacizumab exposure resulted in the upregulation of VEGF-A, -B, -C, and P1GF [50]. Further, in the same study, it was demonstrated that bevacizumab significantly increased migration and invasion in these chronically treated cells [50]. This atoning phenomenon also extends beyond malignant cells, as it was discovered that treating conjunctival neovascularisation with subconjunctival bevacizumab injections can significantly increase the presence of specific cytokines and growth factors in tears [69]. Also, significant increases in a similar cytokine profile in the aqueous humour are evident after intravitreal bevacizumab injections for the treatment of diabetic retinopathy [51].

Future studies should focus on elucidating shared molecular biology pathways that enable cells to defy growth factor inhibition. Following the acquisition of such knowledge, a plausible approach would be to inhibit these internal signalling intermediates, with the intention of preventing cells from inducing compensatory mechanisms.

## Conclusion

In this article, we demonstrated that three UM cells produce VEGF-A and the primary receptor VEGF-R2 in culture. We also showed that the receptor is activated in both a paracrine and a autocrine fashion, which can be inhibited by a non-lethal dose of bevacizumab. Although VEGF-A generally acts as a mitogen and induces proliferation in other malignancies, there are limited studies investigating the consequences of this cascade in UM. Based on the rationale that producing ligands and receptors requires the expenditure of valuable energy resources, it is reasonable to assume that VEGF may play an important role in UM progression, despite the fact that it does not ubiquitously inhibit their functional abilities *in vitro*.

It is evident that even the most promising and conceptually sound pharmaceutical intervention strategies can incur unwanted and unpredictable side effects. The ideology that inhibiting a tumour’s blood supply undeniably conforms with the aforementioned declaration; however, in UM cell lines, abrogating VEGF-A induces secretory changes in factors that can promote the accumulation of macrophages, which are indicators of poor prognosis, and gelatinases that facilitate haematogenous spreading. Inevitably, dual or multiple therapies are likely required to completely sever all potential compensatory avenues that malignant cells may exploit in response to pharmacological insults. Physicians and researchers alike should be forewarned that exogenous treatments can activate countervailing pathways in highly ploidy cells resulting in the paradoxical generation of more aggressive phenotypes.

## Conflicts of Interest

The authors have no conflicts of interest to declare.
